# The kinesin Eg5 inhibitor K858 exerts antiproliferative and proapoptotic effects and attenuates the invasive potential of head and neck squamous carcinoma cells

**DOI:** 10.1007/s10637-022-01238-2

**Published:** 2022-03-21

**Authors:** Alice Nicolai, Samanta Taurone, Simone Carradori, Marco Artico, Antonio Greco, Roberta Costi, Susanna Scarpa

**Affiliations:** 1 Department of Experimental Medicine, Sapienza University of Rome, Viale Regina Elena 324, 00161 Rome, Italy; 2 Department of Sensory Organs, Sapienza University, Viale del Policlinico 155, 00161 Rome, Italy; 3 Department of Pharmacy, “G. d’Annunzio” University of Chieti-Pescara, Via dei Vestini 31, 66100 Chieti, Italy; 4 Istituto Pasteur-Fondazione Cenci Bolognetti, Dipartimento Di Chimica E Tecnologie del Farmaco, Sapienza University of Rome, Piazzale Aldo Moro 5, 00161 Rome, Italy

**Keywords:** Kinesin Eg5, K858, Head and neck cancer, Kinesin KIF11

## Abstract

Our group recently demonstrated that K858, an inhibitor of motor kinesin Eg5, has important antiproliferative and apoptotic effects on breast cancer, prostatic cancer, melanoma and glioblastoma cells. Since high levels of kinesin Eg5 expression have been correlated with a poor prognosis in laryngeal carcinoma, we decided to test the anticancer activity of K858 toward this tumor, which belongs to the group of head and neck squamous cell carcinomas (HNSCCs). These cancers are characterized by low responsiveness to therapy. The effects of K858 on the proliferation and assembly of mitotic spindles of three human HNSCC cell lines were studied using cytotoxicity assays and immunofluorescence for tubulin. The effect of K858 on the cell cycle was analyzed by FACS. The expression levels of cyclin B1 and several markers of apoptosis and invasion were studied by Western blot. Finally, the negative regulation of the malignant phenotype by K858 was evaluated by an invasion assay. K858 inhibited cell replication by rendering cells incapable of developing normal bipolar mitotic spindles. At the same time, K858 blocked the cell cycle in the G2 phase and induced the accumulation of cytoplasmic cyclin B and, eventually, apoptosis. Additionally, K858 inhibited cell migration and attenuated the malignant phenotype. The data described confirm that kinesin Eg5 is an interesting target for new anticancer strategies and suggest that this compound may be a powerful tool for an alternative therapeutic approach to HNSCCs.

## Introduction

Head and neck squamous cell carcinomas (HNSCCs) are a group of extremely aggressive tumors since almost 50% of cases have a survival rate of below 5 years from diagnosis [1, 2]. The high mortality rate of HNSCCs is mainly due to the existence of distant metastases at the moment of diagnosis [3] since the chemotherapy used for metastatic disease often results in low responses [4, 5]. The poor prognosis of HNSCC is also due to the development of multiple primary tumors and to local and regional often inoperable relapses [6, 7]. Therefore, it is important to find additional targets for new drugs directed against these carcinomas, which are the fifth most common cancer in the world [8]. One of these targets should be mitotic kinesins, which are a large family of microtubule-based motor proteins specifically involved in various phases of the mitotic event [9]. These proteins act as molecular motors coordinating cell replication and are particularly overexpressed in highly proliferating tissues, such as tumors, but are hardly expressed in nonproliferating cells [10]. Among these motor kinesins, Eg5/KIF11 contributes to the formation and maintenance of bipolar mitotic spindles during cell division and is consequently expressed almost exclusively in proliferating cells [11]. The upregulation of Eg5 has been correlated with tumorigenesis, since transgenic mice overexpressing Eg5 showed genomic instability and developed several types of malignancies [10]. Conversely, Eg5 inhibition gives rise to monopolar instead of normally bipolar mitotic spindles and consequently determines mitosis arrest in early prometaphase. As a positive aspect, kinesin Eg5 inhibitors do not interfere with other microtubule-dependent processes [12]; for this reason, they have a low toxicity rate and, unlike other anti-microtubule agents, they do not cause neuropathy in patients [13]. For this last characteristic, and because kinesin Eg5 is overexpressed in many cancers [14] and is also predictive of an unfavorable prognosis in breast cancer [15], it can be an interesting target for new antineoplastic compounds directed toward HNSCCs. In fact, a high rate of expression of kinesin Eg5 has been detected in laryngeal squamous cell carcinoma in correlation with the prognostic grade [16].

A compound named monastrol was the first molecule with Eg5 kinesin inhibitory activity to be synthesized [17], which was followed by several analogs [14, 18]. Among these compounds, a small molecule named K858 has been synthesized [19], and over the years, our group has reported significant antineoplastic activity of K858 in preclinical studies on different tumors, such as prostate and breast carcinomas [20–22], melanoma [20, 21] and glioblastoma [23]. In this study, we analyzed the effects of K858 on three HNSCC cell lines with regard to replication, apoptosis and invasion characteristics. We demonstrated that this compound inhibited proliferation, induced apoptosis and reversed the invasive phenotype. The presented data are suggestive of an important *in vitro* anticancer activity of K858 for HNSCCs.

## Materials and methods

### Cell culture and treatment

Three human HNSCC cell lines were utilized: CAL27, SCC-15 (tongue carcinoma) and FaDu (pharynx carcinoma) (American Type Culture Collection, Manassas, VA, USA). These cell lines were grown at 37 °C in RPMI 1640 medium supplemented with 10% fetal calf serum (FCS), 2 mM glutamine and 50 U/ml penicillin–streptomycin. K858 was synthesized, purified (> 96%) and characterized as previously described [20]; it was solubilized in dimethylsulfoxide (DMSO) (Sigma–Aldrich) for a 1 mM stock solution. Control cells were treated with equivalent amounts of DMSO in every experiment.

### Cytotoxicity assay

A 96-well plate was seeded with 5,000 cells per well for 24 h, and then the cells were treated with different concentrations of K858 for 24 and 48 h. The cells were fixed with 50% trichloroacetic acid for 1 h at 4 °C and stained with 0.4% sulforhodamine B in 1% acetic acid for 30 min at room temperature (RT). The excess dye was removed by washing four times with 1% acetic acid. The cells were dissolved in 10 mM Tris (pH 10), and the optical density was measured using a microplate reader at 510 nm.

### Flow cytometry

Cells were treated with 1 μM K858 or equivalent amounts of DMSO for 16 h and then detached with trypsin, washed twice with cold PBS and fixed with 70% ethanol at 4 °C overnight. The cells were then rinsed twice with PBS and incubated with 10 mg/ml RNase (Sigma Aldrich) and 1 mg/ml propidium iodide (Sigma Aldrich) for 2 h at RT in the dark. The phases of the cell cycle were analyzed by a FACS Calibur flow cytometer using the Cell Quest analysis program. The experiment was performed in triplicate for each cell line, and the mean values were calculated.

### Immunofluorescence

The cells were grown on Labteck chamber slides (Nunc) for 24 h and then treated with 1 μM K858 or DMSO for 24 h. The cells were then washed with PBS with Ca/Mg (washing buffer) and fixed with 4% buffered paraformaldehyde (Sigma Aldrich) for 20 min at 4 °C. The cells were incubated in blocking buffer (PBS, 5% FCS, 0.5% Triton) for 30 min at RT and then incubated for 1 h at RT with a primary rabbit polyclonal antibody against beta-tubulin (1:200; Immunological Sciences, Italy). Alternatively, after fixation, the cells were permeabilized with 0.1% Triton for 10 min at RT, incubated with 3% bovine serum albumin (BSA) for 1 h at RT and then incubated at 4 °C overnight with a primary rabbit polyclonal antibody to cyclin B1 with 0.1% BSA (1:200; Elabscience, TX, USA). The cells were washed twice with washing buffer and then incubated with FITC-conjugated secondary anti-rabbit antibody (1:400; Molecular Probes, Oregon, USA) for 1 h at RT. The cells were washed twice with washing buffer and finally stained with Hoechst for 15 min at RT, mounted with ProLong Antifade reagent (Life Technologies) and analyzed by a fluorescence microscope (Olympus BX52). Image acquisition and processing were conducted by IAS 2000 software. Immunofluorescence intensity was quantified using ImageJ v.1.48 software (National Institutes of Health, Bethesda, MD, USA). The densitometry of 10 randomly selected fixed squares from the cytoplasm of single cells was measured and expressed as densitometric units (DUs), and the mean values ± the standard deviations (SDs) were then calculated.

### Western blot

Cell lysates were obtained by incubating the cells in lysis buffer (1% Triton, 0.1% sodium dodecyl sulfate, 150 mM NaCl, 50 mM Tris HCl pH 7.4, 2 mM EDTA) with a protease inhibitor cocktail tablet (Roche Applied Science, Germany) for 30 min at 4 °C. Lysates were centrifuged at 16,000 × g for 15 min at 4 °C, and the supernatants were collected. Protein concentration was evaluated using the Protein Concentration Assay (Bio–Rad Laboratories, CA, USA). Protein samples (50 μg) were separated by 10, 12 or 14% SDS–PAGE and then transferred onto nitrocellulose membranes. Membranes were blocked in 5% nonfat dry milk for 1 h at RT and then incubated with the primary antibodies overnight at 4 °C, washed in Tris-buffered saline with 0.1% Tween 20 and incubated with horseradish peroxidase-conjugated anti-mouse or anti-rabbit IgG (1:5000; Sigma–Aldrich) for 1 h at RT. The filters were then developed by enhanced chemiluminescence (Super Signal West Pico Chemiluminescence Substrate; Thermo Fisher Scientific, MA, USA) using Kodak X-Omat films (Kodak, NY, USA). The primary antibodies used were as follows: rabbit anti-cyclin B1 (1:500; Elabscience, TX, USA); mouse anti-PARP-1 (1:500; Santa Cruz Biotechnology, TX, USA); mouse anti-caspase 8 (1:500; Cell Signaling Technology, MA, USA); mouse anti-caspase 9 (1:500; Cell Signaling Technology); mouse anti-Bcl-2 (1:200; Santa Cruz Biotechnology); rabbit anti-Bax (1:250; Santa Cruz Biotechnology); rabbit anti-E-cadherin (1:1000; GeneTex, CA, USA); rabbit anti-N-cadherin (1:1000; GeneTex); rabbit anti-MMP1 (1:500; Biomol, Italy); rabbit anti-MMP2 (1:1000; GeneTex); rabbit anti-MMP9 (1:700; GeneTex); and rabbit anti-tubulin (1:4000; Immunological Sciences). The experiments were performed in duplicate or triplicate. The bands were quantified using ImageJ v.1.48 software and are expressed as DU.

### Invasion assay

Cell invasiveness was assayed with Matrigel Invasion Chambers (Corning). The cells were detached and resuspended in serum-free medium containing 1 μM K858 or DMSO, and 50,000 cells were plated in the insert chamber, which consisted of a Matrigel membrane with 8 μm pores. Complete medium with 10% FCS was added to the lower chamber to serve as a chemoattractant. After 16 h of culture, the insert was washed with PBS with Ca/Mg and fixed with 100% methanol for 30 min at 4 °C. The insert was then washed twice with PBS with Ca/Mg and stained with hematoxylin for 20 min at RT. The migratory cells were counted with a microscope (Olympus BX52) using an average of 10 fields per insert. Image acquisition was conducted using IAS 2000 software.

### Statistical analyses

All data were processed using one-way analysis of variance, and significance was evaluated using Tukey’s honest post hoc test. The cytotoxicity assays were run at least three times; their statistical analyses and histograms were produced using Graph Pad Prism 5.0.

## Results

### K858 inhibits HNSCC proliferation through mitotic spindle impairment and cell cycle delay in G2/M

**Fig. 1:**
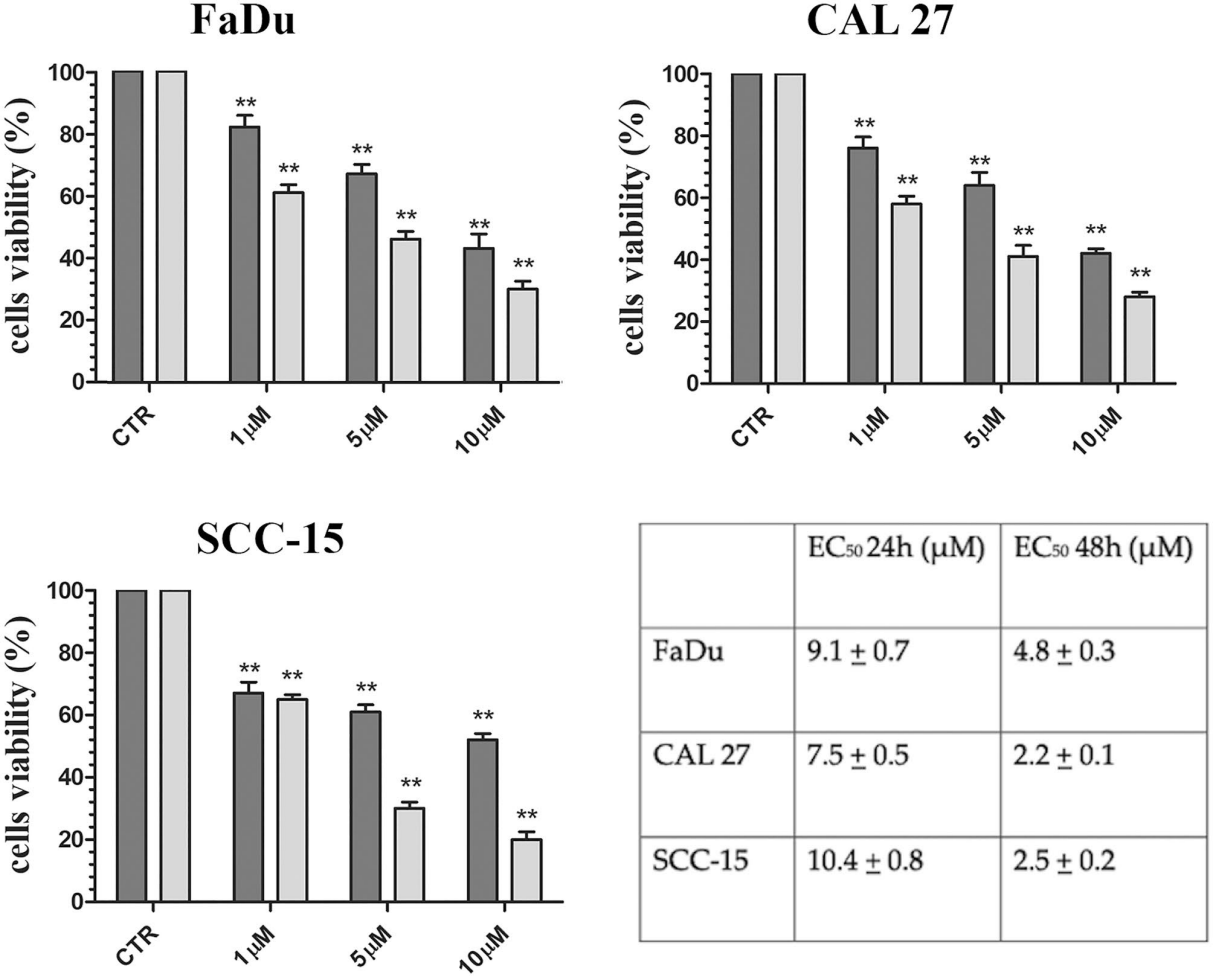
Viability of untreated (CTR) and K858-treated FaDu, CAL27 and SSC-15 cells expressed as percentages of live cells ± SD at 24 h (dark gray) and 48 h (light gray). *p < 0.01; **p < 0.001. The table shows the EC_50_ ± SD at 24 and 48 h

To evaluate the effects of K858 on cell viability and replication, three HNSCC cell lines (FaDu, CAL27 and SCC-15) were treated for 24 and 48 h with 1, 5 and 10 μM K858. The treatment resulted in significant decreases in cell viability in all three HNSCCs at 24 h at the lowest K858 concentration of 1 μM. The cell viability further decreased with increasing concentrations and timing of K858 treatment (Fig. 1). We repeated the time points of K858 concentrations at 1, 10, 20, 30 and 40 μM (data not shown) to calculate the half maximal effective concentrations (EC_50_) at 24 and 48 h for each cell line (Fig. 1).

**Fig. 2:**
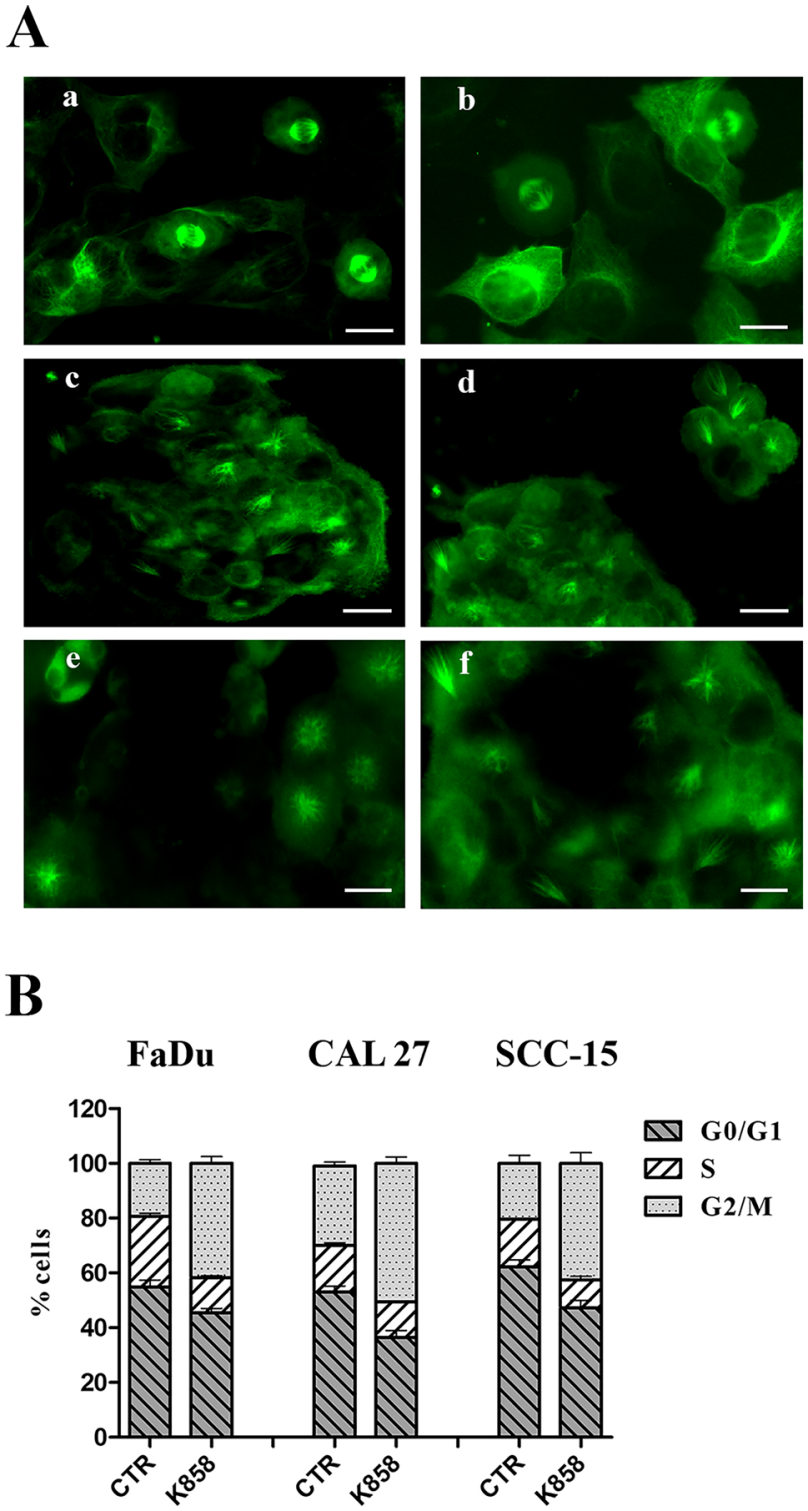
**A** – Immunofluorescence of beta tubulin in CAL27 (**a**) and FaDu (**b**) untreated cells and in CAL27 (**c**), FaDu (**d**) and SCC-15 (**e**–**f**) cells treated with 1 μM K858 for 24 h. Bars: 7 μm (**a**), 5 μm (**b**), 8 μm (**c**-**d**), and 7 μm (**e**–**f**). **B** – Cell cycle analysis by flow cytometry of FaDu, CAL27 and SCC-15 cells untreated (CTR) and treated with 1 μM K858 for 16 h. The histogram shows the means ± SD from three independent experiments

The inhibition of kinesin Eg5 can lead to alteration of tubulin polymerization within the microtubules with the consequent irreversible impairment of the assembly of mitotic spindles; therefore, the effect of K858 on mitotic spindle structure was studied. Immunofluorescent staining of beta-tubulin was performed in all three HNSCC cell lines untreated and treated with 1 μM K858 for 24 h, and then the cells undergoing mitosis were observed with a fluorescence microscope. Each mitotic spindle of untreated HNSCC cells had a normal bipolar shape (Fig. 2A, a-b). In contrast, all mitotic spindles of K858-treated cells had an abnormal monopolar structure, and no bipolar mitotic spindles were detectable (Fig. 2A, c-f).

The inhibition of kinesin Eg5 can determine the impairment of the regular organization of tubulin within the microtubules with a consequent irreversible alteration of the assembly of mitotic spindles. Therefore, the effects of K858 on mitotic spindle shape were studied by immunofluorescent staining of beta-tubulin in all three HNSCC cell lines untreated and treated with 1 μM K858 for 24 h. The structural damage caused by K858 on mitotic spindles probably resulted in abortive mitosis. Therefore, the cell cycles of the three HNSCC cell lines untreated and treated with 1 μM K858 for 16 h were analyzed by flow cytometry. Major changes in the distribution of cells in the different phases of the cell cycle were induced by K858 treatment, with a marked increment of cells in the G2/M phase. Specifically, K858 increased the cell percentages blocked in G2/M from 19.4 to 42% in FaDu cells, from 29 to 50.4% in CAL27 cells and from 20.3 to 42.4% in SCC-15 cells (Fig. 2B).

### K858 upregulates cytoplasmic cyclin B1

**Fig. 3:**
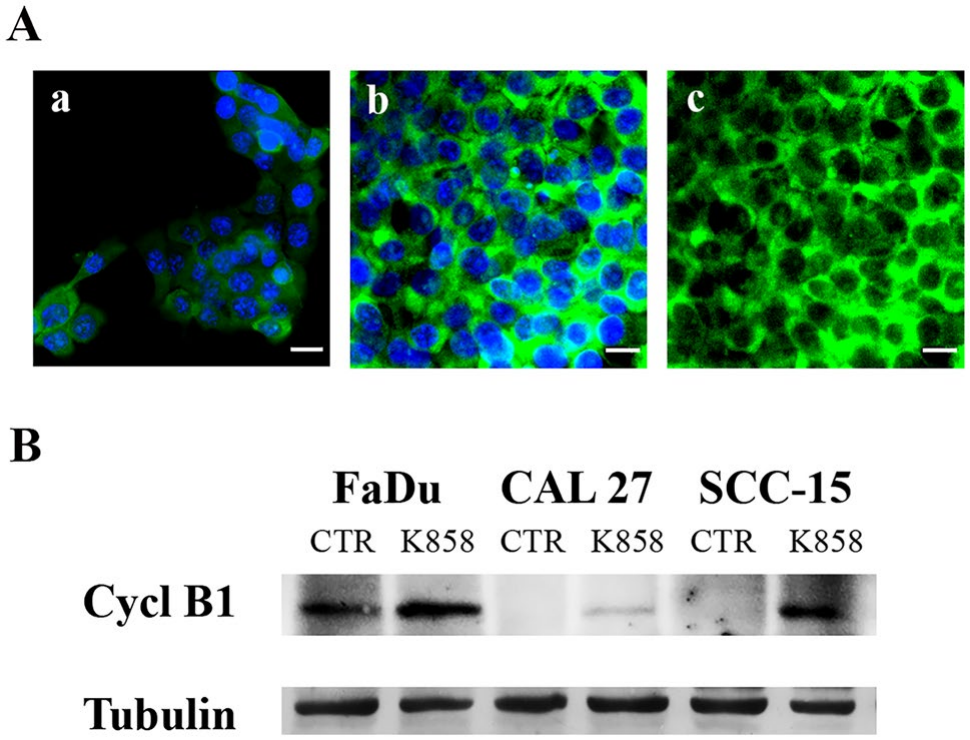
**A** – Immunofluorescence of cyclin B1 in untreated SCC-15 cells (**a**) and SCC-15 cells treated with 1 μM K858 for 24 h shown with (**b**) and without (**c**) nuclear staining. Bar, 7 μm. **B** – Western blot of cyclin B1 and tubulin in untreated (CTR) HNSCC cells and HNSCC cells treated with 1 μM K858 for 24 h

The expression and cellular localization of cyclin B1 were then analyzed since it physiologically increases and then translocates from the cytoplasm to the nucleus during the transition of the cell cycle from S to G2 and then to M phases [24]. We performed cyclin B1 immunofluorescence and found that 1 μM K858 treatment for 24 h resulted in evident increases in cyclin B1 expression in all three HNSCCs. However, cyclin B1 localization was exclusively cytoplasmic, while no translocation in the nucleus was evident (Fig. 3A). The densitometric quantification of cyclin B1 staining resulted in 225 ± 18 DU in untreated cells (Panel a) and 856 ± 41 DU in K858-treated cells (Panel b), showing a 3.8-fold increase in cytosolic cyclin B1 after treatment. In parallel, a Western blot was performed, confirming the upregulation of cyclin B1 in all three HNSCCs after treatment with 1 μM K858 for 24 h (Fig. 3B).

### K858 induces apoptosis

**Fig. 4:**
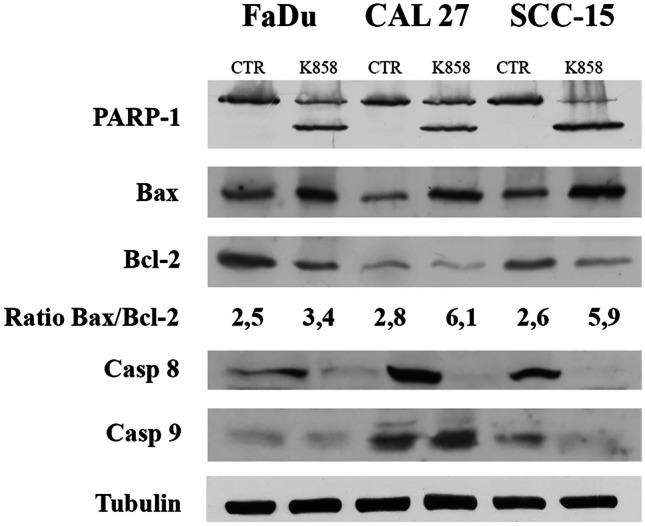
Western blot of PARP-1, Bax, Bcl-2, caspase 8, caspase 9 and tubulin from FaDu, CAL27 and SCC-15 cells untreated (CTR) and treated with 2.5 μM K858 for 24 h. The means from densitometry values of three different experiments of the Bax to Bcl-2 ratio normalized to tubulin are indicated

K858 activity as an inducer of apoptosis was subsequently investigated since a delay or block of the cell cycle can represent a starting point for apoptosis. Therefore, the expression of PARP-1 was evaluated because its cleavage by caspase 3 occurs during the late phase of apoptosis. We found that treatment with 2.5 μM K858 for 24 h induced PARP-1 cleavage in all three HNSCCs (Fig. 4). Two other apoptosis-related proteins, antiapoptotic Bcl-2 and proapoptotic Bax, were evaluated since an increased Bax/Bcl-2 ratio usually indicates apoptosis. We found that Bax was always upregulated and Bcl-2 was downregulated in HNSCCs treated with 2.5 μM K858 for 24 h. The densitometry of each band was measured, and the resulting Bax/Bcl2 ratio significantly increased in all three HNSCCs after treatment (Fig. 4). To better define the pathway of K858-dependent apoptosis, the expression levels of the initiator caspases 8 and 9 were analyzed, and it was found that caspase 8 was cleaved in every cell line after K858 treatment, while caspase 9 was cleaved exclusively in SSC-15 cells (Fig. 4). The cleavage of caspases is indicative of their activation. Based on this result, the apoptosis induced by K858 probably followed the extrinsic pathway conducted by caspase 8 in all three HNSCC samples, while exclusively in SCC-15 cells, the intrinsic pathway was also activated by caspase 9 activation.

### K858 downregulates the malignant phenotype and inhibits cell migration and invasion

**Fig. 5:**
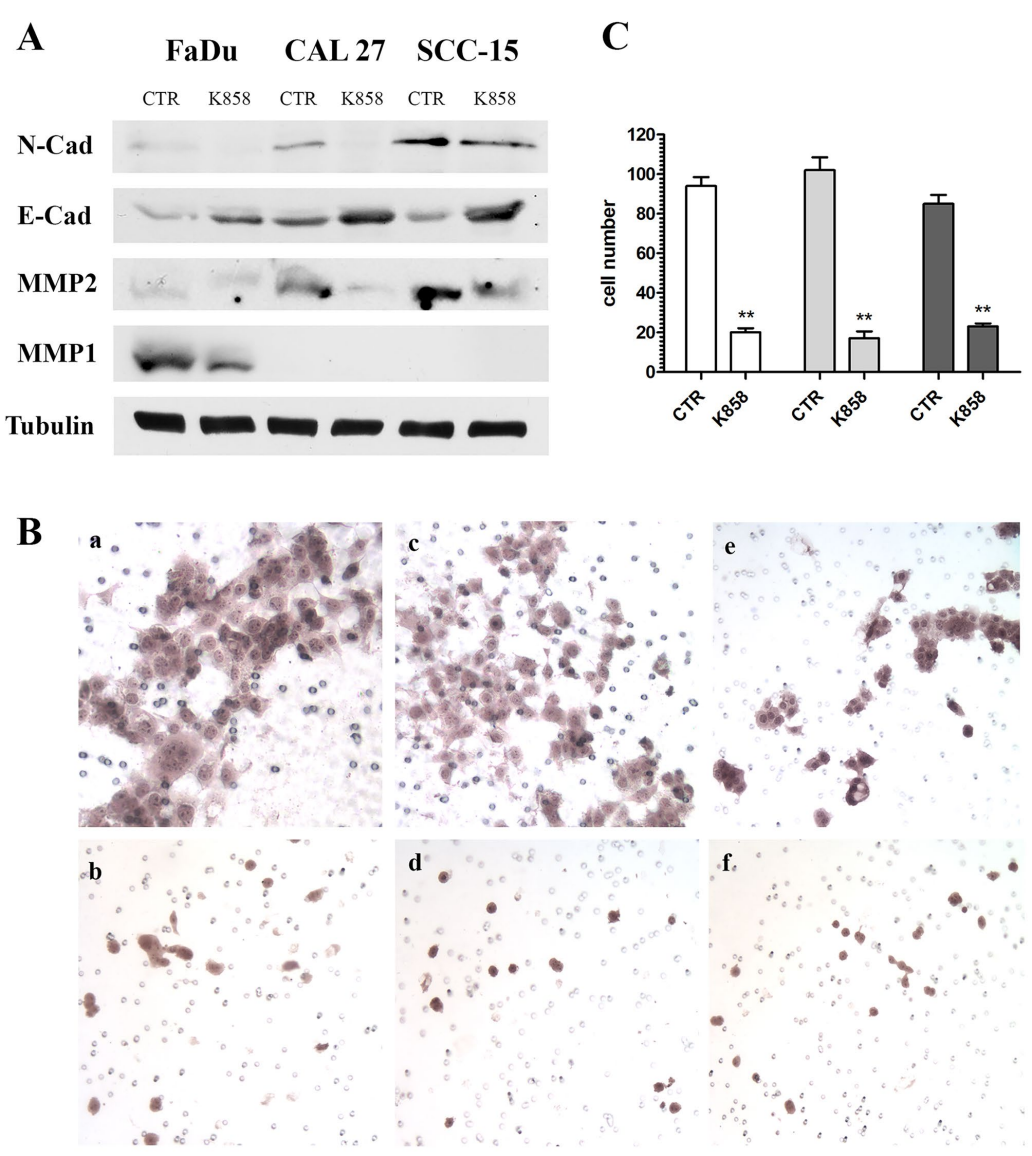
**A** – Western blot of N-cadherin, E-cadherin, MMP2, MMP1 and tubulin from FaDu, CAL27 and SCC-15 cells untreated (CTR) and treated with 1 μM K858 for 24 h. **B** – Matrigel invasion assay of FaDu (**a**-**b**), CAL27 (**c**-**d**) and SCC-15 cells (**e**–**f**) untreated (**a**, **c**, **e**) and treated with 1 μM K858 for 16 h (**b**, **d**, **f**). **C**–Migrated cells in five random fields from three independent experiments were counted, and the means ± SD are reported in the histogram. **p < 0.001

Invasiveness is a peculiar feature of malignant tumor cells and is usually supported by the epithelial-mesenchymal transition (EMT) and by the neo-synthesis of lytic enzymes, in particular matrix metalloproteinases (MMPs) [25]. Therefore, we performed Western blot analysis for two important EMT markers, E-cadherin and N-cadherin, and for three MMPs, MMP1, MMP2 and MMP9. N-cadherin was basally expressed in untreated HNSCCs, and its expression decreased or completely disappeared after treatment with 1 μM K858 for 24 h. E-cadherin was also expressed at baseline levels, and its expression increased in all three HNSCCs when treated with K858 (Fig. 5A). However, CAL27 and SCC-15 cells produced high levels of MMP2, while FaDu cells produced MMP1, and none of the cell lines expressed evaluable levels of MMP9. When the cells were treated with 1 μM K858 for 24 h, both MMP2 and MMP1 were dramatically downregulated (Fig. 5A).

Finally, the degree of malignancy of these tumor cells was quantified by the Matrigel invasion assay, and all three HNSCCs basally showed a high index of motility, while treatment with 1 μM K858 for 16 h was sufficient to abolish the ability of the cells to migrate through the Matrigel (Fig. 5B). The migrated cells were counted, and the results showed that K858 decreased the invasive capacity of FaDu cells by 79%, CAL27 cells by 83% and SCC-15 cells by 73% (Fig. 5C).

## Discussion

All the data showed that K858 was capable of inhibiting proliferation, blocking the cell cycle, inducing apoptosis and attenuating the invasive phenotype of all three HNSCC cell lines.

First, we described that K858 induced an important inhibition of the replication of all three analyzed HNSCCs and that this effect was surely obtained through interference with the assembly of the mitotic spindles. This was demonstrated by the loss of normal bipolarity and the acquisition of an abnormal structure by the mitotic spindles in HNSCC cells treated with K858. In parallel, K858 determined the delay of the cell cycle in the G2 phase, also confirmed by a significant increase in cyclin B1 accumulated exclusively in the cytoplasm. Physiologically, cyclin B1 is synthesized as the cell approaches the G2 phase and is subsequently activated by phosphorylation and translocation from the cytoplasm to the nucleus to allow entry into the M phase and the initiation of mitosis [24]. In K858-treated cells, cyclin B1 expression increased, but it accumulated in the cytoplasm without translocating into the nucleus, with the consequent impairment of its activation: this could have contributed to the interruption of the cell cycle between the G2 and M phases. Normally, when a cell undergoes prolonged prometaphase arrest, it progresses toward apoptosis [26]. In fact, other authors showed that the inhibition of kinesin Eg5 with antisense oligonucleotide in chronic myeloid leukemia cells was able to determine the block of the cell cycle in G2/M and the subsequent death by apoptosis [27]. Accordingly, we observed that K858, after inhibiting mitosis and interrupting the cell cycle in G2, acted as an inducer of apoptosis in all three HNSCCs, as demonstrated by the cleavage of PARP-1 and the increase in the Bax/Bcl-2 ratio. K858 induced apoptosis probably following the extrinsic pathway in all three HNSCCs, as evidenced by the specific cleavage of caspase 8; however, at the same time and exclusively in SSC-15 cells, the intrinsic pathway was also induced, as shown by the cleavage of caspase 9. These data correlate with our previous findings in which K858 was capable of inducing both intrinsic and extrinsic pathways of apoptosis in glioblastoma and breast cancer cells [22, 23].

One of the cellular hallmarks of carcinoma malignancy and aggressiveness is epithelial to mesenchymal transition (EMT), which involves the remodeling of adhesion molecules, with epithelial E-cadherin downregulated and replaced by N-cadherin and/or vimentin [28]. This modification in phenotype makes carcinoma cells able to migrate and invade surrounding healthy tissues more easily and faster [29]. In accordance with the high clinical degree of HNSCC malignancy, we found that all three tumor cell lines basally expressed low levels of E-cadherin and high levels of N-cadherin, confirming an EMT pattern typical of an invasive tumor. We demonstrated that K858 was able to reverse this EMT phenotype, upregulating E-cadherin and downregulating N-cadherin in HNSCC cells. This result is also supported by the report that Eg5 may be involved in the positive regulation of cancer cell migration and development [30].

The clinical behavior of HNSCCs is characterized by high rates of local invasion and distant metastases, and the resulting depth of tumor infiltration and extranodal extension are key parameters to be considered for tumor staging [31]. As expected, all three HNSCC cell lines showed a significant baseline invasiveness index in the extracellular matrix Matrigel, and K858 markedly impaired cell motility and Matrigel infiltration of all three HNSCCs, almost completely preventing their invasive capacity. In this regard, it was previously indicated that knockdown of KIF11 kinesin was able to abrogate the chemotactic migration of breast carcinoma cells [32] and that the allosteric inhibition of Eg5 kinesin by dimethylenastron suppressed pancreatic tumor cell movement and invasion [33].

Another feature of invasive cancer cells is the peculiarity of producing lytic enzymes, such as matrix metalloproteinases, which have long been associated with solid tumor metastases [34]. Indeed, significant synthesis of MMP1 and MMP2 was observed in the analyzed HNSCC cells, which was drastically reduced by K858 treatment. This latter result is particularly interesting because high MMP2 expression has recently been associated with an increased risk of worse overall survival and disease-free survival of HNSCCs [35].

When compared with other anticancer compounds, such as DNA-damaging agents or microtubule drugs, K858 has an important advantageous property because this small molecule blocks the separation of centrosomes, causing the formation of monopolar spindles that lead to mitotic arrest without microtubule reorganization [19]. This peculiarity of K858 can prevent any neurotoxic side effect, as has been shown in human tumor xenografts of mice treated with K858 compared to the same mice treated with paclitaxel [19]. However, the inhibition of KIF11 obtained with two small synthetic molecules different from K858 showed pronounced antitumor activity in xenograft mice with breast cancer without determining any signs of peripheral neuropathy, as is common with other microtubule-targeting anticancer compounds [36].

Some other compounds acting as Eg5 inhibitors are currently utilized as antitumor agents in phase I and II clinical trials, and their good tolerability and lack of neurotoxicity have been reported [37]. When used in monotherapy, these agents demonstrated a limited clinical response, while their effect was more significant when used in combination with other antitumor drugs, resulting in important enhancements of both drug activities [37, 38]. Another interesting point is that K858 induces mitotic cell death exclusively in cancer cells and not in normal nontransformed epithelial cells [19], as our group had previously shown in HaCaT cells and immortalized human keratinocytes [21]; this is explained by the fact that the efficacy of K858 is mainly directed toward cells with a high rate of Eg5 kinesin expression.

Taken together, these data demonstrate that K858 is capable of slowing proliferation, interrupting the cell cycle at the G2 phase, inducing apoptosis, reversing EMT and weakening the invasive phenotype of HNSCC cells. In conclusion, the activity shown by K858 supports its role as a valuable additional tool capable of controlling the malignant growth and progression of HNSCCs.

## Data Availability

All the data presented and the materials used in this study are available on request from the corresponding author.
